# Application of the integrated behavioral model to identify the predictors of toothbrushing practices among primary school children at Bahir Dar city, Ethiopia

**DOI:** 10.1186/s12903-022-02676-3

**Published:** 2022-12-24

**Authors:** Natnael Kebede, Habtamu Wondiye, Lidiya Melkamu, Tadele Fentabil Anagaw, Elias Assefa, Eyob Ketema Bogale, Gebremedhin Hailu, Yirgalem Mohammed, Bezawit Adane

**Affiliations:** 1 Department of Health Promotion, School of Public Health, College of Medicine Health Sciences, Wollo University, Dessie, Ethiopia; 2 Department of Health Promotion, College of Medicine Health Sciences, Bahir Dar University, Bahir Dar, Ethiopia; 3 Department of Health Promotion, College of Medicine Health Sciences, Mizan Tipi University, Tepi, Ethiopia; 4 Department of Health Promotion, College of Medicine Health Sciences, Arsi University, Assela, Ethiopia; 5 Department of Health System and Policy, School of Public Health, College of Medicine and Health Sciences, Wollo University, Dessie, Ethiopia; 6 Department of Epidemiology and Biostatistics, School of Public Health, College of Medicine Health Sciences, Wollo University, Dessie, Ethiopia

**Keywords:** Toothbrushing practices, Integrated behavioral model, Primary school children, Predictors, Ethiopia

## Abstract

Knowing the level of behavioral intention and tooth-brushing practices is crucial for the implementation of the intervention. However, such studies are too limited in Ethiopia. The current study employed a health behavior model to identify predictors that can serve to support primary school children's attitudes, intentions, knowledge, environmental constraints, and practices of tooth brushing. Thus, this study aimed to assess tooth brushing practices and their predictors among primary school children in Bahir Dar city, Ethiopia. An Institutional based cross-sectional study was conducted among primary school children in Bahir Dar city. A multi-stage sampling technique was used to select 610 participants. Data were collected using pre-tested interviewer-administered questionnaires. Questionnaires adapted from items' previous literature were used for integrated behavioral model constructs incorporated with elicitation study results. Data were entered into Epi data and then analyzed by Stata. Descriptive statistics were done. Confirmatory factor analysis was performed to check the convergent validity of the measurement. The Internal reliability of the items was also checked using composite reliability. Multivariable logistic regression was used to predict the role of independent variables in toothbrushing practices. Moreover, path analysis was performed to check the causal effect of integrated behavioral model constructs on toothbrushing practices. The goodness of fit of the final model was checked using the Hosmer and Lemeshow test of best fit with a large *p* value = 0.97 and Area under receiver operating characteristics curve = 0.98. The overall prevalence of the current practice of toothbrushing among the respondents was 45.4%. The prevalence of brushing frequency was 243 (89.01%), 27 (9.89%), and 3 (1.09%) brushed once a day, twice a day, and more than twice a day respectively. Female child's [AOR 3.23, 95% CI 1.48–7.02], mothers' education [AOR 4.6; 95% CI 1.22–17.44], past experience of toothbrushing [AOR 0.042; CI 0.018–0.101], knowledge about tooth brushing practices [AOR 1.3; 95% CI 1.09–1.60], behavioral intention [AOR 2.01; 95% CI 1.74–2.32], experiential attitude [AOR 1.09; 95% CI 1.01–1.17],instrumental attitude [AOR 1.02; 95% CI 1.01–1.03], and descriptive norm [AOR 1.07; 95% CI 1.01–1.14] were predictors of toothbrushing practices. The findings indicate that the practice of toothbrushing practices among primary school students was low. Sex, mother's education, knowledge, intention, experience, experiential attitude, instrumental attitude, and descriptive norm, have significant effects on toothbrushing practices; indicating that the integrated behavioral model showed adequate utility in predicting toothbrushing practices in the study area. School-based toothbrushing practices change interventions such as communication strategy.

## Introduction

Health education and the use of health promotion theories and models are significantly associated with reductions in high-risk disease behaviors, including oral health problems [1]. The integrated behavioral Model (IBM) is the latest formulation of a reason-action approach to predicting and understanding human behavior. A rational approach to action assumes that people behave reasonably under their beliefs. The seven key determinants of behavior are intentions, attitudes, norms, self-efficacy/perceived behavioral control, behavioral beliefs/cost–benefit/outcome expectation, normative beliefs, and control beliefs [2].

According to the model, behavioral intentions are determined by a triple structure. The first is the attitude towards behavior. Many theorists describe attitudes as composed of affective and cognitive dimensions [3]. An experiential attitude or influence is an individual's emotional response to the thought of performing a recommended behavior. People who had a strong negative emotional response to the behavior were less likely to engage, while those who had a strong positive emotional response were more likely to engage. Instrumental attitudes are cognitively based and determined by beliefs about the consequences of behavioral performance [4].

Perceived norms (reflecting social pressure one feels to perform or not perform a particular behavior, what others think one should do, and motivations to comply) may not fully capture normative effects. Perceptions of what other people do in one's social or personal network (descriptive norms) can also be an important component of normative influence [5]. This structure captures the strong social identity in a given culture, which some theorists consider an indicator of normative influence [6]. As mentioned earlier, perceived control depends on the perception of the degree to which various factors make it easy or difficult to perform the behavior [7].

The Integrated Behavioral Model is still primarily applicable to adults or parents of children, as their behavior is usually more logical and rational than that of children. To reduce the child's response burden [8]. Behavioral change-based strategies require the application of effective theoretical evidence-based methods. Integrated Behavioral Mode is one of the most behavioral change models for preventive programs used for public health problems [9].

The toothbrush is the most common method for removing plaque from the oral cavity [10]. Various studies have been made on the toothbrushing habits of children. Investigations have been directed primarily toward the ability of children to perform different methods of toothbrushing, and performance has been determined by the effectiveness of plaque removal [11]. Toothbrushes are the most widely used oral hygiene aids [12]. Brushing twice a day with fluoride toothpaste is one of the most important habits for good oral health. Through Brush day and night activities, children learn about the benefits of good oral hygiene and are taught to brush their teeth twice daily with fluoride toothpaste [13, 14].

Although great achievements have been made in the oral health of the global population, there are still problems in many countries around the world, especially in the poor group in developed and developing countries. Dental caries and periodontal diseases are the most important oral health burdens worldwide. At present, the distribution and severity of oral diseases vary in different regions of the world and within the same country or region. A large number of epidemiological investigations have demonstrated the important role of social behavior and environmental factors in oral diseases and health [15].

The prevalence of recommended tooth brushing behavior has increased in developed countries such as Estonia, Russia, Latvia, Finland, and Flemish Belgium [16]. In India and Indonesia children with the correct habit of brushing have a lower incidence of dental caries compared to children who rarely brush their teeth [17, 18]. Furthermore, an Oral hygiene practice of primary school children in Saudi Arabia Suggested that poor oral hygiene practices are the main risk factor for dental decay among students [19].

Due to poor oral hygiene, dental caries has become a major oral health problem affecting 2.43 billion people worldwide. Even though the WHO is working with countries to develop policies to prevent oral health problems, tooth decay affects an estimated 60–90% of schoolchildren and nearly 100% of adults worldwide. The incidence of tooth decay in low- and middle-income countries is rapidly increasing among adults and children and there will be a huge burden of this health problem in the future without sustainable prevention programs [20]. In Africa, poor oral hygiene is the leading cause of school absenteeism, with children who experience dental pain missing school and performing poorly academically [21]. In Ethiopia, the practice of oral hygiene lacks proper attention and care, and the habit of tooth brushing is very low [22, 23]. Additionally, in Jimma, Angola, and Debre Tabor, Ethiopia the children showed that more than half of the children had adequate knowledge of proper hygiene but only one-third of children experienced tooth brushing [23, 24].

The poor practice of toothbrushing is the major contributing factor to dental caries as a result which can lead to pain, suffering, and reduced quality of life throughout the life cycle. Children with poor oral health miss more school and receive lower grades than those with better oral health, while adults lose more school or work hours for urgent, unplanned dental services [25].

The WHO has identified key strategies for improving oral health including cost-effectiveness has a high and sustainable impact and that includes a mix of preventive population-wide and patient-centered care, with a clear focus on health promotion and empowerment for effective self-care, with a focus on low-income and marginalized populations where the access to oral health care is limited [26].

Toothbrushing practices are one element of quality of life. Primary school students are an appropriate age for the foundation for the longstanding life of tooth brushing behavior, knowing the level of their behavioral intention and tooth brushing practices is crucial for the implementation of the intervention They use many different models for understanding behavior change and designing successful interventions. Among these integrative behavioral model is the most influential model. However, there is no study done using the integrative behavioral model on toothbrushing behavior in previous studies in Ethiopia. Since studies conducted in Ethiopia focus only on knowledge and Practices, this study employed a health behavior model to identify potential variables that can serve to support primary school children’s attitudes, intentions, knowledge, environmental constraints, and practices of toothbrushing practices. It also filled the gap well in the study area. Hence, this study aimed to determine toothbrushing practices and associated factors among Bahir Dar city primary school students using IBM as a conceptual framework.

This finding will provide supportive evidence for health professionals in addressing problems related to behavioral, normative, and control beliefs about the tooth brushing behavior of primary school children. The research findings also help policymakers, program managers, and political leaders plan, monitor, and evaluate the program. It also helps researchers as baseline information for further investigation.

## Methods and materials

### Study setting, design, and period

An institution-based cross-sectional study was conducted from February 28 to March 28, 2021, in Bahir Dar City among students attending primary school. The city is located in northwestern Ethiopia and is the 5^th^ largest city in Ethiopia with an estimated population of 750,991. Bahir Dar City is the capital of the Amhara land region. The city is located approximately 565KM northwest of Addis Ababa and at an elevation of 1840 m above sea level.

Regarding Education services, there are a total of 19,344 primary school students in Bahir Dar city. The city comprises 14 governmental primary schools with a total of students 11,831 and the city also comprises 26 private primary schools with a total of students 7513—the age of students who reached their age for elementary school program (7 years and above).

### Population and sample

All students attending primary school in Bahir Dar city were the target population for this study. The selected eight primary school students attending Bahir Dar city, who fulfilled the inclusion criteria were considered as the study population.

All students who were present at randomly selected primary schools during data collection were included in the study. Students who were unable to respond because of illness were excluded from the study.

The sample size was calculated using Epi info version 7.1; by taking the proportion (43%) of toothbrushing practices from a cross-sectional study in Addis Ababa [27]. By considering the Level of confidence = 95%, (Z α/_2_ = 1.96), D (margin of error) = 0.05, design effect 1.5, and population size 19,344 the sample size was calculated as:

$${\text{Where}}\,{\text{n}} = \frac{{\left( {{\text{Z}}\upalpha /2} \right)^{{2}} \,{\text{P}}\left( {{1} - {\text{P}}} \right)}}{{{\text{d}}^{2} }} \to {\text{n}} = \frac{{\left( {{1}.{96}} \right)^{{2}} \left( {0.{43}} \right)\,\left( {{1} - 0.{43}} \right)}}{{\left( {0.0{5}} \right)^{{2}} }} = 554$$

Thus, the total sample size was = 554 + 10% non-response = 610.

A multi-stage sampling technique was employed to select the study units. After governmental and private primary schools were included in the study 20% of the schools were randomly selected. There were 6194 total numbers of students in the selected schools. All grades were taken as a stratum and a final simple random sampling method was used to determine the participants. The students' rosters for each grade level were made available from each of the schools. The total sample size was proportionally allocated to each school based on the number of students in each grade. Finally, simple random sampling using a computer random number generator was used to select study participants in each grade using the student roster from the register to select 610 students. Of the 14 governmental primary schools 3 were randomly selected and in 26 private primary schools, 5 were randomly selected.

### Study variables and measurement

The dependent variable in this study was tooth-brushing behavior. The independent variables of the study were Socio-demographic factors (age, sex, father and mother's educational status, father and mother's Employment status, and the number of children).

Intention, experiential attitude, instrumental attitude, injunctive norm, descriptive norm, perceived behavioral control, self-efficacy, experience (Habit), environmental constraints, and knowledge.

Direct measures of constructs are essential for two reasons. First, direct measures are usually more strongly associated with intentions and behaviors than indirect measures. The associations between the direct measures and behavioral intention are used to indicate the relative importance of attitude, perceived norm, and perceived control in explaining or predicting a given behavior. It is important to demonstrate these associations before analyzing indirect measures. Second, indirect measures should be associated strongly with direct measures to ensure that appropriate beliefs were included in the indirect measures and that composite beliefs (behavioral, normative, and control) are adequate measures of the respective IBM constructs. Once this is demonstrated, indirect measures are of most interest for the development of communication-based interventions. Behavioral, normative, and control beliefs help us understand what drives behaviors and provide foci for intervention messaging using multiple communication modes or channels [28].

*Toothbrushing practices* Toothbrushing practice was assessed by the self-reported status of Brushing teeth at least once per day in the last week [29].

*Intention* an indication of individual readiness/willingness/ to practice tooth brushing and how much effort they are planning to exert, to practice toothbrushing four items were measured using a 5-point bipolar Likert scale, summed up to go the score with higher scores indicating a higher intention to practice toothbrushing.

*Experiential attitude* is defined as an individual’s emotional response toward performing the recommended behavior. Directly it was measured.

With four items five-point SDS items were summed to obtain the score with higher scores indicating a favorable attitude and indirectly measured by behavioral belief five items using a five-point Likert scale summing up across to obtain the score with higher scores indicating a favorable attitude.

*Instrumental attitude* This is based on the cognitive outcome evaluation of tooth brushing behavior. The direct measure of attitude toward performing the practices was obtained using five-point SDS four items were summed to obtain the score with a higher score indicating a favorable attitude and indirectly measured by the behavioral belief of ten items using a five-point Likert scale and multiplied by the corresponding outcome evaluation of ten items. The score was computed by summing the products of beliefs and evaluations with higher scores indicating a favorable attitude.

*Injunctive norm* refers to the social pressure from others that an individual feels. Directly measured by four items with 5 points Likert scale, summing up to obtain the score with a higher score indicating a highly influential. Indirect measurement was obtained by having participants rate normative beliefs concerning whether different sources of influence approve the participant to practice tooth brushing behavior and the participant's motivation to comply with those sources. The score was calculated by computing the products of four items of normative belief and their motivation to comply and summing up to obtain an overall score, a higher score indicating approval of the behavior.

*Descriptive norm* refers to whether the referents perform tooth brushing behavior. Four items were Directly measured based on the belief that most people perform the behavior by using a 5-point Likert scale summed up to obtain the score with higher scores indicating more practice of toothbrushing. Four items were indirectly measured by normative belief summed up to obtain the score with a higher score indicating more to practice toothbrushing.

*Perceived control* one’s perception of the degree to which various environmental factors make it easy or difficult to carry out the behavior. Perceived control was directly measured by the overall measure of perceived control over the behavior by four items with five-point SDS and summed up to obtain the score with higher scores indicating that the factor was under control and indirectly measured by control belief five items multiplied by five perceived power items. The scores were computed by summing the products of control beliefs and perceived power values with a higher score indicating that the factor was under control.

*Self-efficacy* is one’s degree of confidence in the ability to perform the behavior even in the face of various obstacles or challenges. They are directly measured by 5-point Likert scales containing three items and indirectly measured by 5-point Likert scales containing five items and summed up to obtain the score with a higher score indicating better confidence in the practice of toothbrushing.

*Knowledge* The study participants were asked 11 knowledge questions and, the total score was obtained for each respondent summed up to obtain the score a high score indicated better knowledge.

### Data collection instrument

An elicitation study was conducted on 20 Shimbit primary school children in Bahir Dar city, before developing closed-ended questions [8]. Elicitation study is a critical step in the application of an integrated behavioral model. The participants were asked to answer 12 open-ended questions to elicit their behavioral beliefs, normative beliefs, and control beliefs about toothbrushing practices. Often very different behavioral, normative, efficacy, and control beliefs affect intentions to engage in different behaviors. IBM is to conduct open-ended elicitation interviews to identify relevant behavioral outcomes, referents, facilitators, and barriers for each particular behavior and target population under investigation.

### Data collection tools and techniques

An interviewer-administered questionnaire was used to collect data. Eight data collectors and two supervisors were recruited and data were collected in the class setting. All data collectors were oriented for half a day before the data collection period by the principal investigator on the objectives of the study and how to administer the questionnaires, the issues of verbal consent, the writer not to participate in the study, and how to assist the respondents on questions that were not clear during data collection. Questionnaires were adapted from items used in previous studies and IBM constructs incorporated with elicitation study results [30, 31]. The questionnaires included; socio-demographic variables (8 items), the practice of toothbrushing 5 items), knowledge about tooth brushing (12 items), Intention (4 items), Experiential attitude (9 items), instrumental attitude (24 items), Injunctive norm (12 items), Descriptive norm (8 items), perceived control (14 items), self-efficacy (8 items) and environmental constraints (2 items).

### Statistical analysis

Data were checked for completeness and consistency. Data were entered into Epi data version 3.02 and exported to Stata version 14.1 for analysis. Descriptive statistical analysis such as frequency and percentage for categorical variables. Logistic regression was performed to determine whether the constructs of IBM could be associated with toothbrushing practices. For all statistical significance tests, the cut-off value set was *p* < 0.05 with a Confidence interval of 95%. Those variables whose *p* values are less than 0.25 during the simple binary logistic regression analysis were fitted to the final multivariable binary logistic regression model to adjust for potential confounders. The odds ratio was used to observe the strength of the association between toothbrushing practices and each significant independent variable. Path analysis was performed to check the causal effect of IBM constructs on toothbrushing practices. The goodness of fit of the final model was checked using the Hosmer and Lemeshow test of best fit with a large *p* value = 0.97 and Area under receiver operating characteristics (ROC) curve = 0.98.

### Data quality assurance

The questionnaire was prepared in English and translated into the Amharic version. Finally, it was back-translated into English by another person to ensure consistency. Pretests were performed on 5% [31] of the study population in Felege Abay primary schools in Bahir Dar City. After the pre-test, the necessary amendment was made. One day of training was given to the data collectors and supervisors on the questionnaire and data collection process. Close supervision was performed by the principal investigator and supervisors throughout the data collection period. The collected data were checked for completeness and consistency. To increase the data quality double data entry to the Epi data was considered. To determine the face validity of the questionnaire, the collected views and comments of advisors and experts in the field of health promotion were taken. Confirmatory factor analysis was conducted to check the convergent validity of the measurements. Based on this analysis, Factor loadings and Eigenvalue of intention (all), experiential attitude (all), instrumental attitude (dropping two items), injunctive norm (all), descriptive norm (all), perceived control (all), and self-efficacy (all) are greater than 0.4 and 1 respectively. The Kaiser–Meyer–Olkin Measure of Sampling Adequacy ranged from 0.74-to 0.94 and Bartlett's Test of Sphericity was significant.

Reliability analysis was conducted to check the internal consistency of the measurement of this study using composite reliability and Cronbach's alpha (α).

### Confirmatory factor analysis

Confirmatory factor analysis was conducted to check of convergent validity of the measurement of this study using factor loadings and Eigenvalue. Based on this analysis, Factor loadings and Eigenvalue of intention (all), experiential attitude (all), instrumental attitude (dropping two items), injunctive norm (all), descriptive norm (all), perceived control (all), and self-efficacy (all) is greater than 0.4 and 1 respectively. Kaiser–Meyer–Olkin Measure of Sampling Adequacy range from 0.74 to 0.94 and Bartlett’s Test of Sphericity is significant (Table 1).

**Table 1:** Confirmatory factor analysis for Application of the integrated behavioral model to identify the predictors of toothbrushing practices among primary school children at Bahir Dar city, Ethiopia, 2021

Construct	Items	Definition	Factor loadings	Eigenvalue
Intention	q301	Question301	0.8626	2.77129
	q302	Question302	0.8716	
	q303	Question303	0.7871	
	q304	Question304	0.8050	
Experiential attitude	q309	Question309	0.7699	2.51505
	q310	Question310	0.7009	
	q311	Question311	0.7409	
	q312	Question312	0.6214	
	q313	Question313	0.7042	
Instrumental attitude	Q31828	Question318*328	0.7236	5.04601
	Q31929	Question319*329	0.8288	
	Q32030	Question320*330	0.8006	
	Q32131	Question321*331	0.8444	
	Q32232	Question322*332	0.8116	
	Q32333	Question323*333	0.7090	
	Q32434	Question324*334	0.3004	
	Q32535	Question325*335	0.3499	
	Q32636	Question326*336	0.7244	
	Q32737	Question327*337	0.7636	
Injunctive norm	Q34246	Question342*346	0.8263	3.17442
	Q34347	Question343*347	0.9671	
	Q34448	Question344*348	0.9610	
	Q34549	Question345*349	0.7956	
Descriptive norm	q354	Question354	0.8662	2.73710
	q355	Question355	0.7995	
	q356	Question356	0.8588	
	q357	Question357	0.7811	
Perceived control	Q36267	Question362*367	0.8511	3.26406
	Q36368	Question363*368	0.6100	
	Q36469	Question364*369	0.8459	
	Q36570	Question365*370	0.8810	
	Q36671	Question366*371	0.8221	
Self-efficacy	q375	Question375	0.7691	2.84114
	q376	Question376	0.7201	
	q377	Question377	0.7828	
	q378	Question378	0.7228	
	q379	Question379	0.7719	

## Findings

### Socio-demographic characteristics

A total of 601 primary school students participated in Bahir Dar city with a response rate of 98.5%. The mean age of the respondents was 13 (± 0.064). Three hundred forty-six (57.57%) of the study participants were female. Concerning the educational status of their family, 171 (28.45%) of the mothers were able to read and write and 105 (17.47%) were unable to read and write. Of the fathers, 200 (33.28%) had College and above education and 159 (26.46%) of the fathers were able to read and write. About 259 (43.09%) mothers and 233 (38.77%) of the fathers of the students were housewives and merchants respectively. Regarding the number of children in the family, the highest proportion was 200 (33.28%) who had more than three children, followed by 188 (31.28%) with two children, 162 (26.96%) with three children, and 51 (8.49%) one child (Table 2).

**Table 2:** Participants' socio-demographic characteristics among primary school children, Bahir Dar city, Ethiopia, April 2021

Variable	Category	Frequency	Percent
School type	Government	347	57.74
	Private	254	42.26
Child’s sex	Male	255	42.43
	Female	346	57.57
Number of children in the family	One child	51	8.49
	Two child	188	31.28
	Three child	162	26.96
	More than three child	200	33.28
Father’s job	Government employee	197	32.78
	Merchant	233	38.77
	Farmer	48	7.99
	Others	123	20.47
Mother’s employment status	Housewife	259	43.09
	Merchant	176	29.28
	Governmental employee	131	21.80
	Others	35	5.82
Fathers’ education	Unable to read and write	44	7.32
	Able to read and write	159	26.46
	Elementary school	66	10.98
	Secondary school	63	10.48
	Grade 12 complete	69	11.48
	College and above	200	33.28
Mothers’ education	Unable to read and write	105	17.47
	Able to read and write	171	28.45
	Elementary school	54	8.99
	Secondary school	38	6.32
	Grade 12 complete	67	11.15
	College and above	166	27.62

### Knowledge and practice of toothbrushing practices

The mean toothbrushing knowledge score of the respondents was found to be 7.23 (± 2.08) with a range of 2 to11. Most (95.51%) of the participants had ever heard about toothbrushing and 47.4% (CI 0.434–0.514) of respondents were aware of electronic toothbrushes. Of the respondents who heard about toothbrushing 153 (26.66%), 146 (25.44%), 113 (19.69%), 81 (14.11%), 63 (10.98%), 12 (2.09%), and 6 (1.05%) of the respondents got the information from dentists, television, internet, school, family, friends, and others respectively.

Regarding participant’s toothbrushing practice, most of the respondents (87.52%) had ever brushed their teeth, 273 (45.42%) currently practiced toothbrushing at least once per day, and 363 (60.40%) had experience toothbrushing practices. The prevalence of brushing frequency was 243 (89.01%), 27 (9.89%), and 3 (1.09%) brushed once a day, twice a day, and more than twice a day respectively. The majority (56.77%) of the study participants preferred toothbrushing in the morning session (Table 3).

**Table 3:** toothbrushing practices among primary school children, Bahir Dar city, Ethiopia, April 2021

	Variables	Frequency	Percent
Have ever brushed your teeth	Yes	526	87.52
	No	75	12.48
Do you brush your teeth within the last week daily	Yes	273	45.42 (CI 0.414–0.494)
	No	328	54.58
Do you brush your teeth before last week	Yes	363	60.40 (CI 0.564–0.642)
	No	238	39.60
How many times per day do you brush your teeth	Once a day	243	89.01
	Twice a day	27	9.89
	More than twice	3	1.09
When do you brush your teeth	In the morning	155	56.77
	After eating	10	3.66
	Before I go to bed	78	28.57
	In the morning and Before I go to bed	30	11.00
Practice of toothbrushing	Yes	273	45.42
	No	328	54.58
Access to toothpaste in our town	Yes	595	99.00
	No	6	1.00
A program that is working to make students aware of tooth brushing in primary school	Yes	215	35.77
	No	386	64.23

### Environmental constraints

Most 595 (99%) of the respondents said access to toothpaste in their town and 386 (64.23%) of the respondents said no program was working to make students aware of tooth brushing in their school.

### Descriptive statistics for the components of the integrated behavioral model

Descriptive statistical analysis was performed to measure the mean score of IBM components. Intention, instrumental attitude, injunctive norm, descriptive norm, and self–efficacy had mean scores of 13.51 (SD = 4.8), 172.78 (SD = 51.04), and 56.95 (SD = 31.01), 13.56 (SD = 4.58), and17.88 (SD = 5.32) respectively. The mean score of experiential attitude was 15.54 (SD = 5.25) which approached the maximum value of the experiential attitude sum score. Perceived control had a low mean score of 55.96 (SD = 33.76) (Table 4).

**Table 4:** Descriptive statistics for the components of the integrated behavioral model of toothbrushing practices among primary school students, Bahir Dar city, Ethiopia, April 2021

Variables	No of items	Min	Max	Mean	SD
Intention	4	4	20	13.51	4.84
Experiential attitude	5	4	20	15.54	5.25
Instrumental attitude	10	45	250	172.78	51.04
Injunctive norm	4	4	100	56.95	31.01
Descriptive norm	4	4	20	13.56	4.58
Perceived control	5	5	125	55.96	33.76
Self-efficacy	5	5	25	17.88	5.32

### Correlation between direct and indirect IBM constructs

IBM construct measures (indirect EA, IA, IN, DN, PBC, and SE) have a strong relationship with their corresponding global measures (direct EA, IA, IN, DN, PBC, and SE), (r = 0.82, *p* < 0.001), (r = 0.85, *p* < 0.001), (r = 0.80, *p* < 0.001), (r = 0.79, *p* < 0.001), (r = 0.57, *p* < 0.001), and (r = 0.57, *p* < 0.001) respectively, indicating that the three beliefs (behavioral beliefs, normative beliefs and control beliefs) which were identified by the elicitation study were adequately captured their corresponding overall measures (Table 5).

**Table 5:** Correlation between direct and indirect measures of IBM model among primary school students, Bahir Dar city, Ethiopia, April 2021

DEA	DIA	DIN	DDN	DC	DS	IEA	IIA	IIN	IDN	IC	IS	
DEA	1.0000											
DIA	0.8265**	1.0000										
DIN	0.7501**	0.7921**	1.0000									
DDN	0.7084**	0.7291**	0.8139**	1.0000								
DC	0.6931**	0.6906**	0.7345**	0.7582**	1.0000							
DS	0.5297**	0.6179**	0.5587**	0.4986**	0.5285**	1.0000						
IEA	0.8163**	0.8316**	0.7055**	0.6511**	0.6342**	0.5696**	1.0000					
IIA	0.8137**	0.8494**	0.8210**	0.7368**	0.7385**	0.5653**	0.7943**	1.0000				
IIN	0.6736**	0.7317**	0.7996**	0.7894**	0.7158**	0.4993**	0.6583**	0.7641**	1.0000			
IDN	0.6414**	0.6542**	0.7290**	0.7873**	0.7602**	0.5070**	0.6067**	0.6982**	0.7038**	1.0000		
IC	0.5145**	0.5070**	0.5167**	0.5059**	0.5707**	0.3978**	0.5091**	0.5345**	0.4887**	0.4699**	1.0000	
IS	0.6995**	0.6895**	0.6840**	0.7010**	0.6773**	0.5703**	0.6353**	0.6981**	0.6436**	0.6473**	0.5349**	1.0000

*DEA* direct experiential attitude, *DIA* direct instrumental attitude, *DIN* direct injunctive norm, *DDN* direct descriptive norm, *DC* direct control, *DS* direct self-efficacy, *IEA* indirect experiential attitude, *IIA* indirect instrumental attitude, *IIN* indirect injunctive norm, *IDN* indirect descriptive norm, *IC* indirect control, *IS* indirect self-efficacy

**Significant at *p* < 0.001

### Factors affecting tooth brushing behavior

Multivariable logistic regression was performed to determine the effect of independent variables on toothbrushing practices. In this model, variables with a *p* value < 0.25 were taken and analyzed together by multivariable logistic regression. The confounding factors were adjusted using multivariable logistic regression models. After controlling for the effects of potentially confounding variables using multivariable logistic regression, grade level, child's sex, mothers' education, the experience of toothbrushing, knowledge, behavioral intention, experiential attitude, instrumental attitude, and descriptive norm were found to be significantly associated with toothbrushing practices at *p* value < 0.05.

Female participants were three times more likely to perform toothbrushing practice than male participants [AOR 3.23, 95% CI 1.48–7.02]. Those students whose mother’s educational status was secondary school and above were 60% more likely to perform toothbrushing practice than those whose mothers were unable to read and write [AOR 4.6; 95% CI 1.22–17.44]. Students who had no experience with toothbrushing were 4% less likely to perform toothbrushing practice than those who had experience in toothbrushing practices [AOR 0.042; CI 0.018–0.101]. With a positive unit change in the knowledge sum score, the odds of toothbrushing practice increased by 30% [AOR 1.3; CI 1.09–1.60]. A positive unit change in intention to brush teeth led to an increase in toothbrushing practice by two times [AOR 2.01; CI –2.32]. One unit increase in experiential attitude led to a 9% increase in toothbrushing practice [AOR 1.09; CI 1.01–1.17]. a positive unit change in instrumental attitude led to a 2% increase in tooth brushing practice [AOR 1.02; CI 1.01–1.03]. A positive unit change in descriptive norm led to a 7% increase in toothbrushing practice [AOR 1.07; CI 1.004–1.144] (Table 6).

**Table 6:** Factors affecting toothbrushing practices of Bahir Dar city primary school students, Ethiopia, April 2021

Variables		Tooth brushing practice		COR	AOR	*P* value	95% CI	
		Yes	No				Lower	Upper
Age				1.1	1.0	0.872	0.752	1.274
Child’s sex	Male	93	162	RC	RC	RC	RC	RC
	Female	180	166	1.9	3.23	0.003*	1.481	7.022
Fathers’ education	unable to read and write	11	33	RC	RC	RC	RC	RC
	Able to read and write	57	102	1.7	1.1	0.929	0.094	13.292
	Elementary school	32	34	2.8	.9	0.946	0.057	14.446
	secondary school and above	173	159	3.3	.9	0.979	0.085	11.029
Mothers’ education	unable to read and write	27	78	RC	RC	RC	RC	RC
	Able to read and write	59	112	1.5	2.7	0.124	0.766	9.143
	Elementary school	13	41	0.9	1.3	0.797	0.207	7.784
	secondary school and above	174	97	5.2	4.6	0.024*	1.218	17.446
Experience in tooth brushing	No	20	218	0.03	0.04	0.000**	0.018	.101
	Yes	253	110	RC	RC	RC	RC	RC
Knowledge				1.7	1.3	0.004*	1.09	1.607
Behavioral intention				2.02	2.01	0.000**	1.74	2.32
Experiential attitude				1.4	1.09	0.024*	1.01	1.17
Instrumental attitude				1.04	1.02	0.000**	1.01	1.03
Injunctive norm				1.04	1.01	0.052	0.99	1.02
Descriptive norm				1.3	1.07	0.038*	1.004	1.144
Perceived control				1.03	1.007	0.094	0.998	1.015
Self-efficacy				1.3	1.056	0.067	0.996	1.120

RC-reference category *Significant at *p* < 0.05; **significant at *p* < 0.001

### Causal path analysis of tooth brushing behavior

**Fig. 1:**
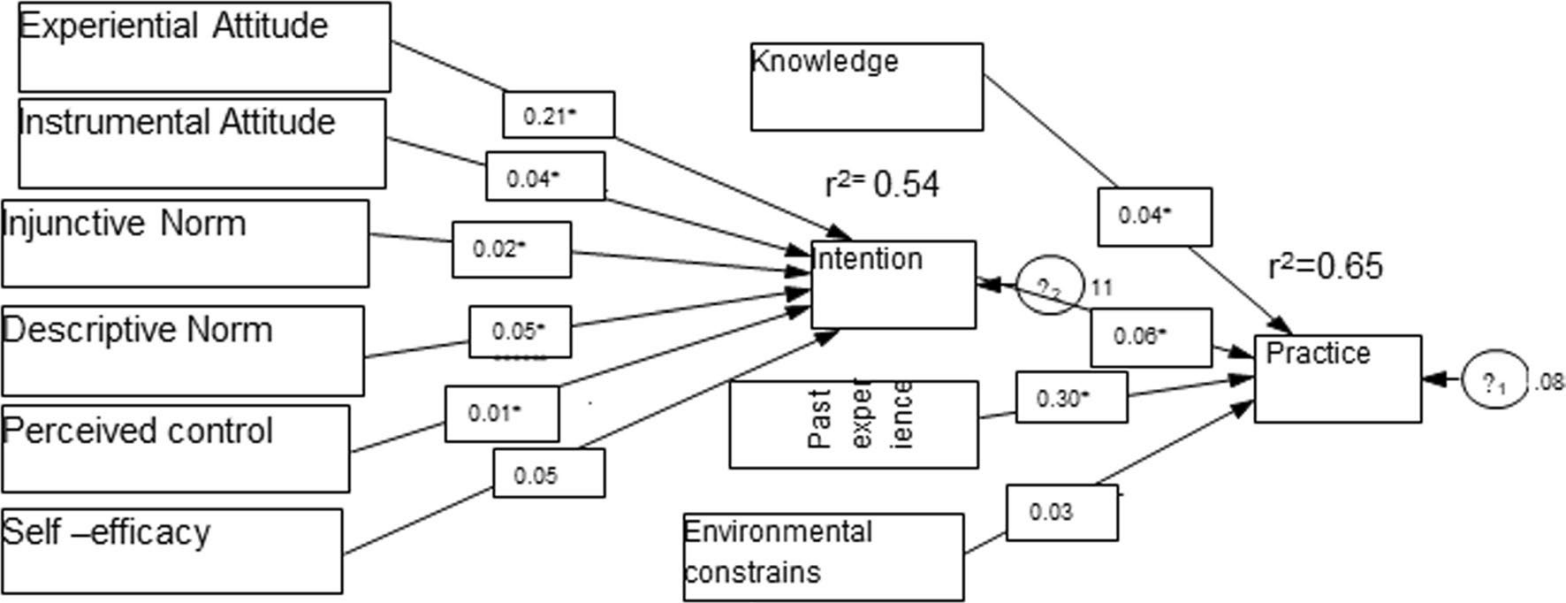
Path model of tooth brushing behavior among grade 5th–8th primary schools students, Bahir Dar city, Ethiopia, April 2021

Intention to toothbrushes was assessed using experiential attitude, instrumental attitude, injunctive norms, descriptive norm, perceived control, and self-efficacy as exogenous variables. In this model, 54% (Adjusted R2 = 0.54) of the variance was explained. Experiential attitude, instrumental attitude, Injunctive Norm, and Perceived control have a positive significant association with behavioral intention (*p* < 0.05) with path coefficients of 0.21, 0.04, 0.02, and 0.01 holding all other relevant variable constants respectively. Knowledge, behavioral intention, and experience of toothbrushing practice were used as exogenous variables to have a significant positive association with toothbrushing practices (*p* < 0.05) with path coefficients of 0.04, 0.06, and 0.30 respectively. Indirect effects were shown through experiential attitude (β = .012, *p* < 0.001), instrumental attitude (β = .002, *p* < 0.001), injunctive norm (β = .001, *p* < 0.001), and perceived control (β = .001, *p* < 0.05) of toothbrushing practices. The Comparative Fit Index (CFI) (0.96), Tucker–Lewis index (TLI) (0.92), and root mean square residual (SRMR) (0.024) proved that the model used to predict behavioral intention and IBM constructs showed acceptable model fit indices (Fig. 1).

## Discussion

In the present study, toothbrushing practices and associated factors among Bahir Dar city primary school students using IBM as a conceptual framework were assessed. The overall prevalence of the current practice of toothbrushing among the respondents was 45.4% (CI 0.414–0.494). This result is in agreement with the findings of 43% of primary school students in Addis Ababa [27]. However, it was found to be higher than the prevalence reported in a study conducted among primary school students in Bahir Dar city, Ethiopia which was 14.3% in 2016 [32]. This was lower than the prevalence reported in a study conducted in Iran which was 56.5% [18]. These differences might be due to the study settings (community or institution and rural or urban), study time, educational status, socioeconomic background status, and differences among the study participants.

In this study, 60.40% (95% CI 0.564–0.642) of respondents had previous experience in toothbrushing practices. This result was lower than that reported in a study in Indonesia which was 97.1% [33]. The difference might be due to educational exposure to toothbrushing practices and socio-economic backgrounds.

In this study, knowledge was significantly associated with toothbrushing practices, consistent with the study done in Gondar, in which good knowledge is the determinant factor for toothbrushing practices [29]. This indicated that providing actual information and increasing the knowledge of the students about toothbrushing practices needs to be incorporated into oral health educational programs to enhance toothbrushing practice. In the present study, 47.4% (CI 0.434–0.514) of respondents were aware of electronic toothbrushes but a much higher study done in India showed that about 96% of the participants were aware of electronic toothbrushes [34]. These discrepancies might be due to the study settings, and technological advancements.

According to the correlational analysis, there was a positive relationship between indirect and direct measurements of the integrated behavioral model. From this, the commonly held salient beliefs extracted from attitudes perceived norms, and perceived behavioral control toward toothbrushing tooth brushing practices were well explained and explored through indirect constructs of the integrated behavioral model. This is consistent with the suggestion of the integrated behavioral model principles in which there is a positive relationship between indirect measurements and their corresponding direct measurements of an integrated behavioral model [8]. This implies that intervention can be designed on the salient beliefs identified during the elicitation study; thus, by influencing the direct measures of IBM, the intention of toothbrushing practices can be increased.

In this study, knowledge, intention, and Past experiences of toothbrushing practices were significantly linked to toothbrushing practice, as suggested by IBM [8]. When a person has a strong intention to perform practice tooth brushing, knowledge about the tooth brushing practice and past experiences of tooth brushing strongest predictors of tooth brushing practice. This indicated that IBM showed adequate utility in predicting toothbrushing practices in the study area since the model assumption is in line with the study findings.

The present study revealed that only mothers' education determined the toothbrushing practice but fathers' education did not play a critical role in determining toothbrushing practices among participants. In contrast, a previous study involving students showed that higher parental education plays a significant role in overall toothbrushing practices [35, 36]. This implies that the prediction of attitude, perceived norm, and perceived behavioral control is not different among the various categories of other socio-demographic characteristics of participants.

### Strengths and limitations of the study

The present study has several strengths, which account for toothbrushing practices to be predicted based on the IBM constructs which may show how much intention could be translated into the practices. Moreover, the strength of this study was conducted entirely based on IBM which provided multiple health behavior factors such as knowledge, environment, and experience (habit) were considered. The elicitation study explored salient beliefs in designing a culturally appropriate survey instrument to measure IBM constructs.

This study had some limitations, since the study design is a cross-sectional study type, it may provide poor prediction and understanding of previous behavior because the time order of constructs IBM and practices cannot be separated in time. A prospective study design is recommended when IBM is used as a conceptual framework to measure the intention of behavior and the behavioral performance at two separate points in time; however, due to a lack of resources and time, the current study did not employ this type of research design. In addition, it may be affected by social desirability bias since the study was self-reported. It should be considered that these statements might differ from actual ones. Children’s oral hygiene needs to be evaluated through clinical examination.

## Conclusions

The findings of this study indicated that the practice of toothbrushing behavior among primary school students is considerably low. Experiential attitude, instrumental attitude, injunctive norm, and perceived behavioral control had significant effects on intention to brush teeth Knowledge, intention, and having experience have significant factors in toothbrushing practices, indicating that IBM indicates adequate utility in predicting tooth brushing behavior in the study area. Socio-demographic variables such as sex, and maternal education were significant predictors of toothbrushing practices. School-based toothbrushing practices change interventions such as Communication strategy and research will be important. In particular, such interventions should give due emphasis to enhancing students' intention to tooth brushing behavior, attitudinal changes, addressing barriers to tooth brushing behavior, and creating positive social pressure from significant others. It is necessary to encourage parents' regular tooth-brushing behavior and promote educational activities through dentists, healthcare providers, and pediatrics.

## Data Availability

All the necessary data are included in the manuscript. An English version data collection tool and detailed operational definitions of the outcome variable are accessible on a reasonable request from the corresponding author.

## Abbreviations

IBM: Integrated behavioral model
PBC: Perceived behavioral control
SDS: Semantic differential scale
TPB: Theory of planned behavior
WHO: World Health Organization
